# Visualization of the distribution of nanoparticle-formulated AZD2811 in mouse tumor model using matrix-assisted laser desorption ionization mass spectrometry imaging

**DOI:** 10.1038/s41598-020-72665-5

**Published:** 2020-09-23

**Authors:** Shoraku Ryu, Mayu Ohuchi, Shigehiro Yagishita, Tatsunori Shimoi, Kan Yonemori, Kenji Tamura, Yasuhiro Fujiwara, Akinobu Hamada

**Affiliations:** 1 Division of Molecular Pharmacology, National Cancer Center Research Institute, Tokyo, Japan; 2 Department of Medical Oncology and Translational Research, Graduate School of Medical and Pharmaceutical Sciences, Kumamoto University, Kumamoto, Japan; 3 Department of Breast and Medical Oncology, National Cancer Center Hospital, Tokyo, Japan

**Keywords:** Cancer, Drug discovery, Oncology, Nanoscience and technology

## Abstract

Penetration of nanoparticles into viable tumor regions is essential for an effective response. Mass spectrometry imaging (MSI) is a novel method for evaluating the intratumoral pharmacokinetics (PK) of a drug in terms of spatial distribution. The application of MSI for analysis of nanomedicine PK remains in its infancy. In this study, we evaluated the applicability of MALDI-MSI for nanoparticle-formulated drug visualization in tumors and biopsies, with an aim toward future application in clinical nanomedicine research. We established an analytic method for the free drug (AZD2811) and then applied it to visualize nanoparticle-formulated AZD2811. MSI analysis demonstrated heterogeneous intratumoral drug distribution in three xenograft tumors. The intensity of MSI signals correlated well with total drug concentration in tumors, indicating that drug distribution can be monitored quantitatively. Analysis of tumor biopsies indicated that MSI is applicable for analyzing the distribution of nanoparticle-formulated drugs in tumor biopsies, suggesting clinical applicability.

## Introduction

Nanoparticle-based drug delivery systems are promising tools for enhancing drug delivery into tumors. To date, 12 nanomedicines in oncology have been approved by the U.S. Food and Drug Administration^1^. Selective delivery to tumors is thought to occur via enhanced permeability and retention (EPR) effects^2^ due to the enhanced porous vasculature of tumors. However, EPR effects exhibit significant variability both between and within tumor types. Many intrinsic factors in tumors are known to influence EPR effects, such as the nature of the vascular system, properties of the stroma, and macrophage infiltration, among others^3^. These factors can vary with tumor size, location, type, and among patients, which can impact the delivery of different drugs in a tumor even with the same type of nanoparticle.

Conventional imaging technologies have been used to evaluate the delivery of nanomedicines in tumors. The most frequently used methods are label-based imaging technologies, which can be used both in vitro and in vivo^4–8^. However, labeling with an imaging agent carries the risk of altering the chemical structure of the parent drug or nanomedicine or disrupting its pharmacologic activity, thus necessitating cautious evaluation^9^. Matrix-assisted laser desorption/ionization (MALDI)–mass spectrometry imaging (MSI) is a new type of imaging technology that enables mapping the distribution of a drug as well as its metabolites in tissues without labeling the drug. MALDI-MSI has been used in recent years to assess small-molecule drug pharmacokinetics (PK), namely PK imaging, in pharmaceutical development^10,11^. MSI also has been used for visualizing the distribution of nanomedicines in tissues by tracking nanomaterials or nanoformulated chemical agents^11–17^. However, reports of the application of MSI in nanomedicine PK imaging, especially in tumors, remain limited^12,17^.

AZD2811 nanoparticles are polyl-d,l-actide(PLA)-poly(ethylene glycol) (PEG) ylated nanoparticles approximately 100 nm in size. The encapsulated payload is AZD2811, an aurora B kinase inhibitor (also known as AZD1152 hydroxyquinazoline pyrazol anilide; AZD1152-hQPA), and the active moiety of the phosphate pro-drug AZD1152/barasertib, which was in clinical development in solid tumors and haematological cancers^18–20^. Aberrant expression of aurora B kinase leads to genomic instability and aneuploidy and was identified in many human cancers^21–23^. AZD2811 showed clinical efficacy in acute myeloid leukemia (AML)^19^; however, due to the inconvenience of administration in AML and dose-limiting myelosuppression in solid tumors, AZD1152 did not proceed further in development. In an attempt to overcome these issues, nanoparticle-formulated AZD2811 was developed^12^.

In the present study, we evaluated the applicability of MALDI-MSI to analyze nanoparticle-formulated AZD2811. The intratumoral distribution of nanoparticle-formulated AZD2811 was visualized using three preclinical xenograft models (MDA-MB231, HCC1954, and PC14). Correlation between MSI signal intensity and liquid chromatography-tandem mass spectrometry (LC–MS/MS) quantitation was confirmed. Moreover, MSI analysis of tumor needle biopsy specimens was carried out with the aim of facilitating clinical application. The results indicate that MALDI-MSI is a suitable tool for imaging analyses of nanoparticle formulations in both preclinical and clinical applications. A comparison of local histologic features with drug distribution as analyzed by high spatial resolution MSI was also attempted.

## Results

### MALDI-MSI visualization of AZD2811 and nanoparticle-formulated AZD2811

**Figure 1:**
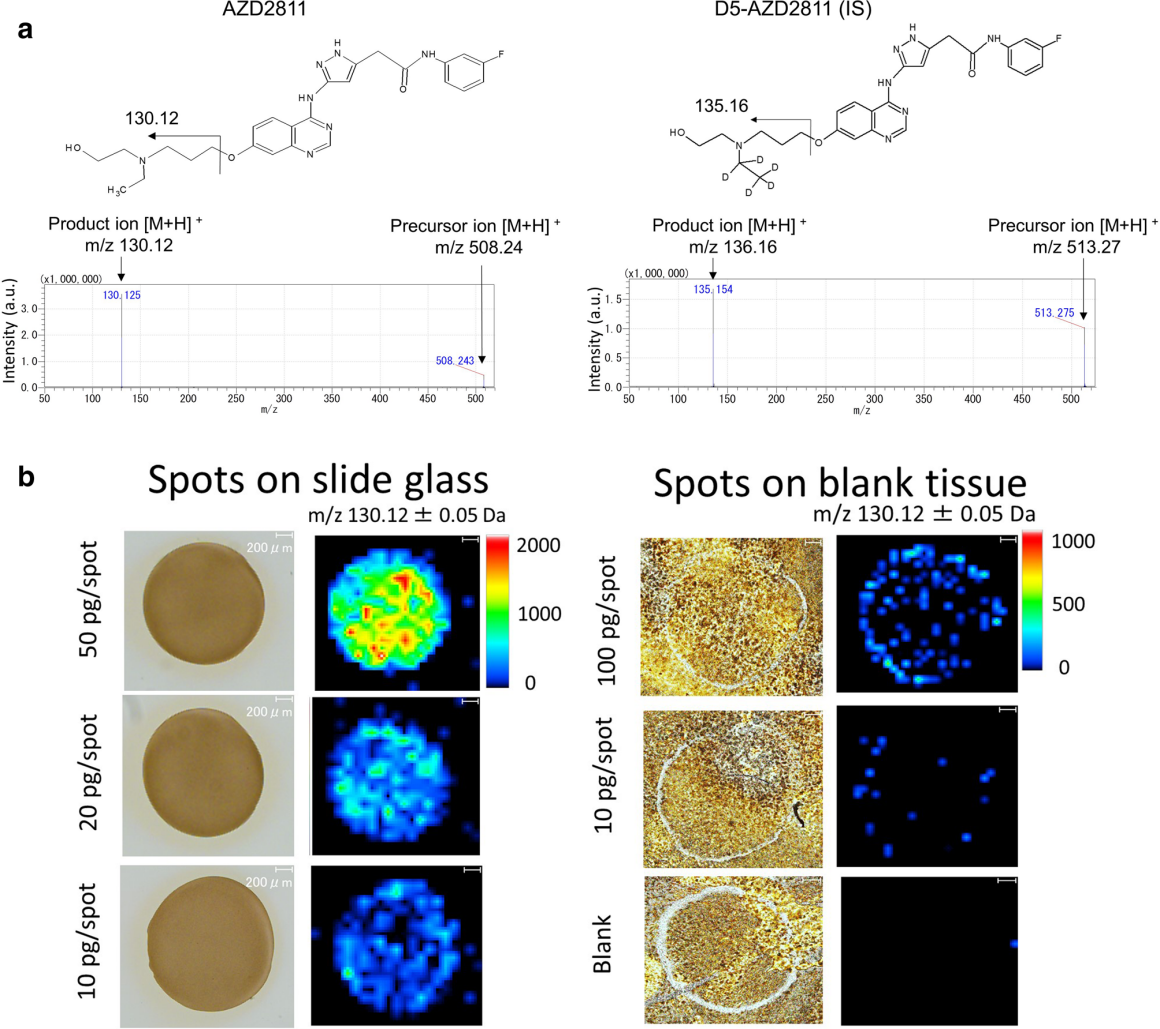
MS/MS spectra and images of AZD2811 standard nanoparticles with optimized MALDI-MSI analysis. (**a**) Chemical structure and MS/MS spectra of AZD2811 (*m/z* 130.12 ± 0.05 Da) and D5-AZD2811 (IS, *m/z* 135.15 ± 0.05 Da) for MSI analysis. (**b**) MSI analysis of nanoparticle-formulated AZD2811 standard spots on a slide glass and blank (untreated MDA-MB231) tumor section. No signal was detected from blank tissue, indicating good specificity. Scale bar: 200 μm. Color bar of MSI images: absolute intensity (a.u.: arbitrary unit).

First, we compared the sensitivity of AZD2811 detection using two different matrixes (α-CHCA and DHB) and acids (FA and TFA) with free AZD2811 standard solution at a concentration of 500 pg/µL spotted on slide glass. CHCA and 0.1% TFA exhibited the best sensitivity and were thus chosen for sample preparation (Fig. S1a). The same evaluation was also performed for D5-AZD2811, which was used as an internal standard (IS). The chemical structures and MS/MS spectra of AZD2811 and D5-AZD2811 are shown in Fig. 1a.

We conducted both MS and MS/MS analysis on the free AZD2811 standard solution spotted on blank tumor section (untreated MDA-MB231 tumor). For MS analysis, we found an endogenously derived peak (m/z 508.30) very near to the target peak of AZD2811 (m/z 508.24). This yielded false positive detection in blank tumor and off-spot area, indicating a low specificity for MS analysis. In contrast, we confirmed the specificity of the product ion (*m/z* 508.24 → 130.12) in MS/MS analysis, as no peak at *m/z* 130.12 was detected from the blank spot or the off-spot area on the blank tumor (Fig. S1b).

We then spotted AZD2811 nanoparticle standard solution on slide glass and blank tumors to confirm the sensitivity and specificity for detecting nanoparticle-formulated AZD2811 via MS/MS analysis in MSI. On the slide glass, AZD2811 nanoparticles showed a concentration-dependent manner and a good sensitivity that higher signal intensity was observed comparing with free AZD2811 at the same concentration (Fig. S1c). On the blank tumor tissue, the ion intensities of the nanoparticle standard spots decreased compared with the standard spots on slide glass at the same concentration (Fig. 1b, Table S1). Ion suppression induced by endogenous components in tissue might be one of the reasons for the observed intensity reduction. Therefore, a simultaneous MSI analysis of D5-AZD2811 was conducted to verify the heterogeneous tissue-derived ion suppression. Moreover, LC–MS/MS was used to validate MSI analysis of tumor samples. The optimized parameters for MSI and LC–MS/MS are shown in Tables S2 and S3.

### MSI analysis of the distribution and PK of AZD2811 in xenograft tumors

MDA-MB231 human triple-negative breast cancer cells (adenocarcinoma), HCC1954 Her2-positive breast cancer cells (ductal carcinoma), and PC14 lung adenocarcinoma cells were evaluated. We analyzed the intratumoral distribution of AZD2811 in cell line xenograft models on days 4, 9, and 14 after administration of AZD2811 nanoparticles on days 1 and 3. The total AZD2811 concentration (including both released and encapsulated AZD2811) in plasma and two consecutive tumor sections adjacent to the section for MSI was evaluated by LC–MS/MS analysis.

**Figure 2:**
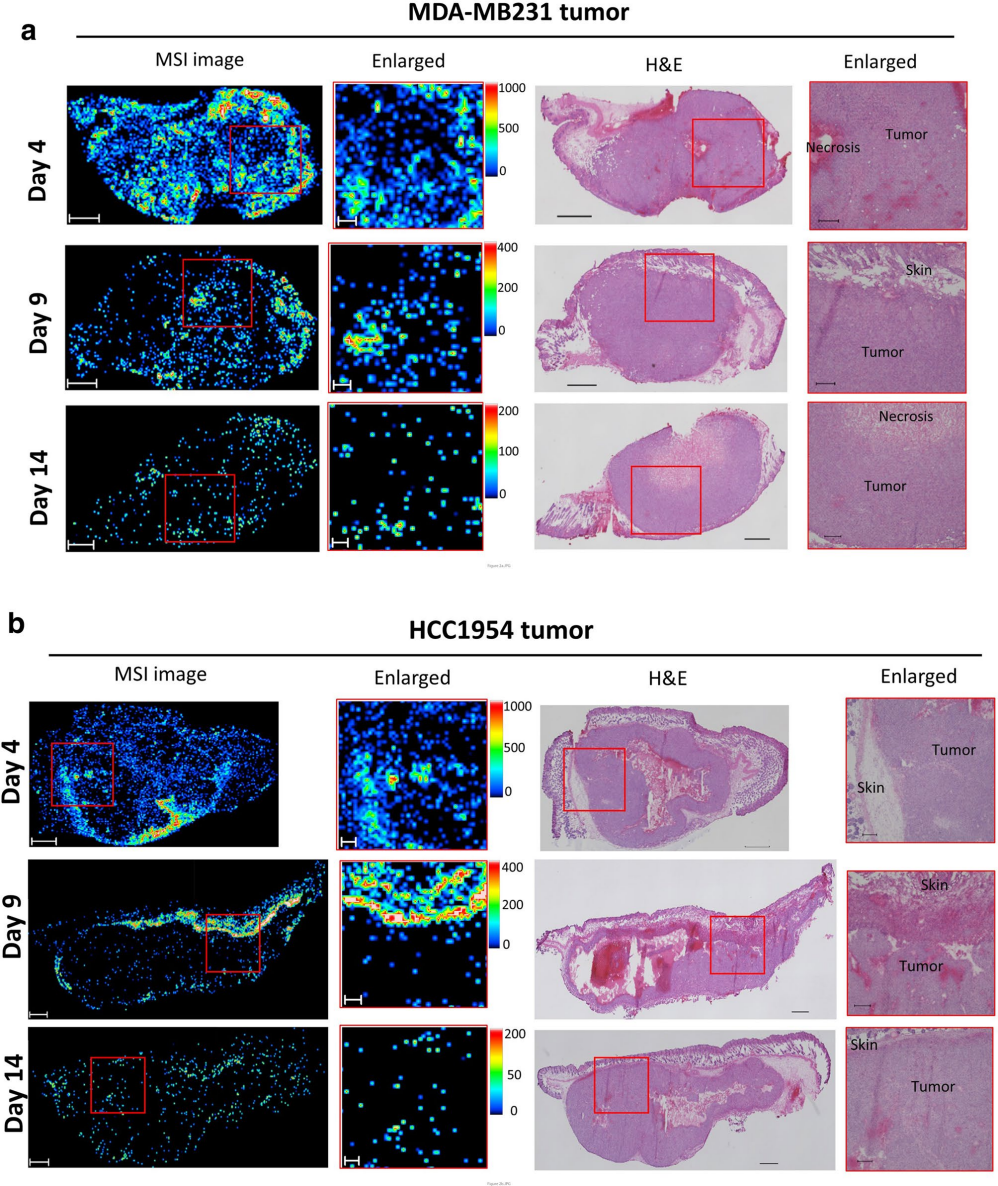

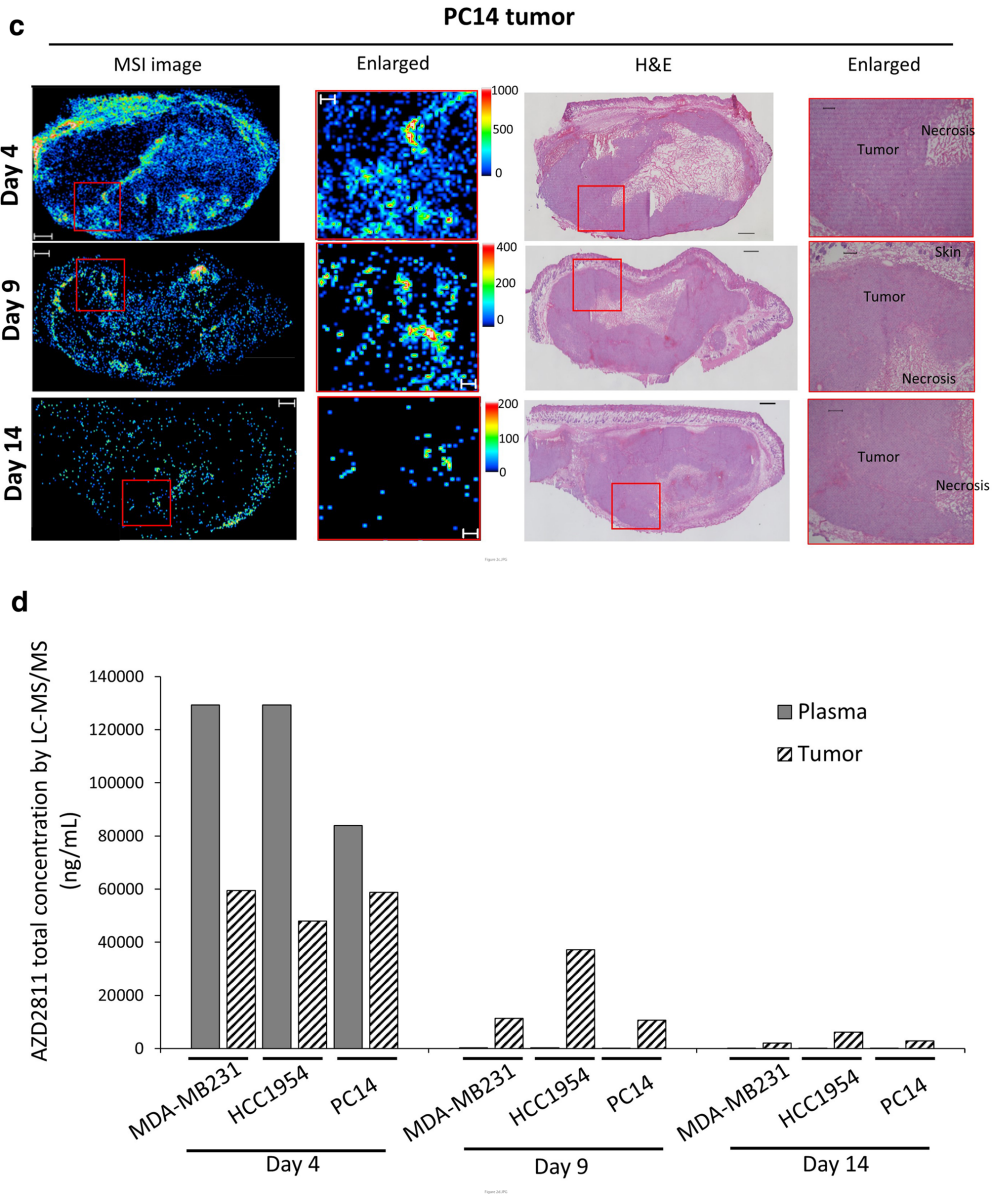
Heterogeneous AZD2811 distribution in three tumors. (**a**) MDA-MB231, (**b**) HCC1954, and (**c**) PC14 cells were inoculated into the flank of mice. MDA-MB231 and HCC1954 were inoculated into the bilateral flank of the same mouse. Tumors were harvested on days 4, 9, and 14 after administration of AZD2811 nanoparticles on days 1 and 3 at a dose of 25 mg/kg (i.v.). Tumors for MSI analysis were obtained from one mouse at each time point. MSI images of AZD2811 (*m/z* 130.12 ± 0.05 Da) (left panels), enlarged MSI images of the red outlined squares (right panels), and the corresponding H&E images are shown. AZD2811 exhibited heterogeneous distribution in tumors. Scale bars: 1000 μm for whole tumor and 250 μm for enlarged images. Spatial resolution for MSI analysis: 50 μm. Color bar of MSI images: absolute intensity (a.u.: arbitrary unit). (**d**) Total AZD2811 concentration in tissues from three tumor types and plasma, as determined by LC–MS/MS analysis.

In MSI analysis, AZD2811 exhibited a heterogeneous distribution, with high signal intensity detected on day 4 in three tumors. On day 9, the AZD2811 mean intensity decreased but still remained in some regions of the tumors. On day 14, AZD2811 was detected in ‘hotspots’ in each tumor. By comparing enlarged MSI with the corresponding H&E images, we found that AZD2811 was distributed very heterogeneously even in relatively homogeneous areas based on H&E images (Fig. 2a–c). The total AZD2811 concentration as determined by LC–MS/MS analysis decreased over time in the tumors as well as plasma. However, the concentration was higher in tumors than in plasma on days 9 and 14 in three tumors, indicating a slower clearance in tumors (Fig. 2d).

In plots of the mean MALDI-MSI signal intensity per unit of area (a.u./mm^2^) versus the average AZD2811 concentration (pg/mm^2^) quantified by LC–MS/MS in two consecutive tissue section , good linearity and R^2^ value was observed for each tumor type. This indicates there is a good agreement between MSI ion response and drug amount indicating that the signal intensity from MSI can depict the relative drug abundance in tissues. It should be noted that the slopes of the plots for the three tumor types differed, and the correlation between all samples decreased (Fig. S2a, the upper panel). After normalization with IS (D5-AZD2811), the disparities between tumor types were not compensated for (Fig. S2a, left of the lower panel). The MSI images of IS showed homogeneous distribution within each section which indicates a relatively uniform intra-tumoral ion suppression for AZD2811 (Fig.S2b). However, IS signal responses differed between tumor types indicating different ion suppression levels in different tumor types (Table S4). A concept of tissue extinction coefficient (TEC) was proposed in the previous studies. TEC was calculated by comparing the mean intensity of a standard on the tissue and on the bare substrate, which has been used to compare the ion suppression effects between different whole organs, regions in highly heterogeneous organs, and different MSI methods^24,25^. We calculated TEC by dividing the on-tissue IS mean intensity to the off-tissue mean intensity (Table S4). Then we plotted AZD2811 mean intensity by normalization with TEC to the drug concentration in each sample and found an improvement in signal response consistency among tumor types (Fig. S2a, right of the lower panel). The results indicate TEC is a useful scaling factor when comparing between different tumor types.

### Application of MSI for analysis of PC14 tumor biopsies and relationship to histopathologic features

In clinical treatment of advanced cancer, collection of tumor tissue via surgery is often not indicated, and biopsy specimens are usually collected. To verify the usefulness of MSI analysis of biopsy specimens, we examined repeat biopsy specimens collected together with whole tumors using the PC14 xenograft model after administration of AZD2811 nanoparticles.

**Figure 3:**
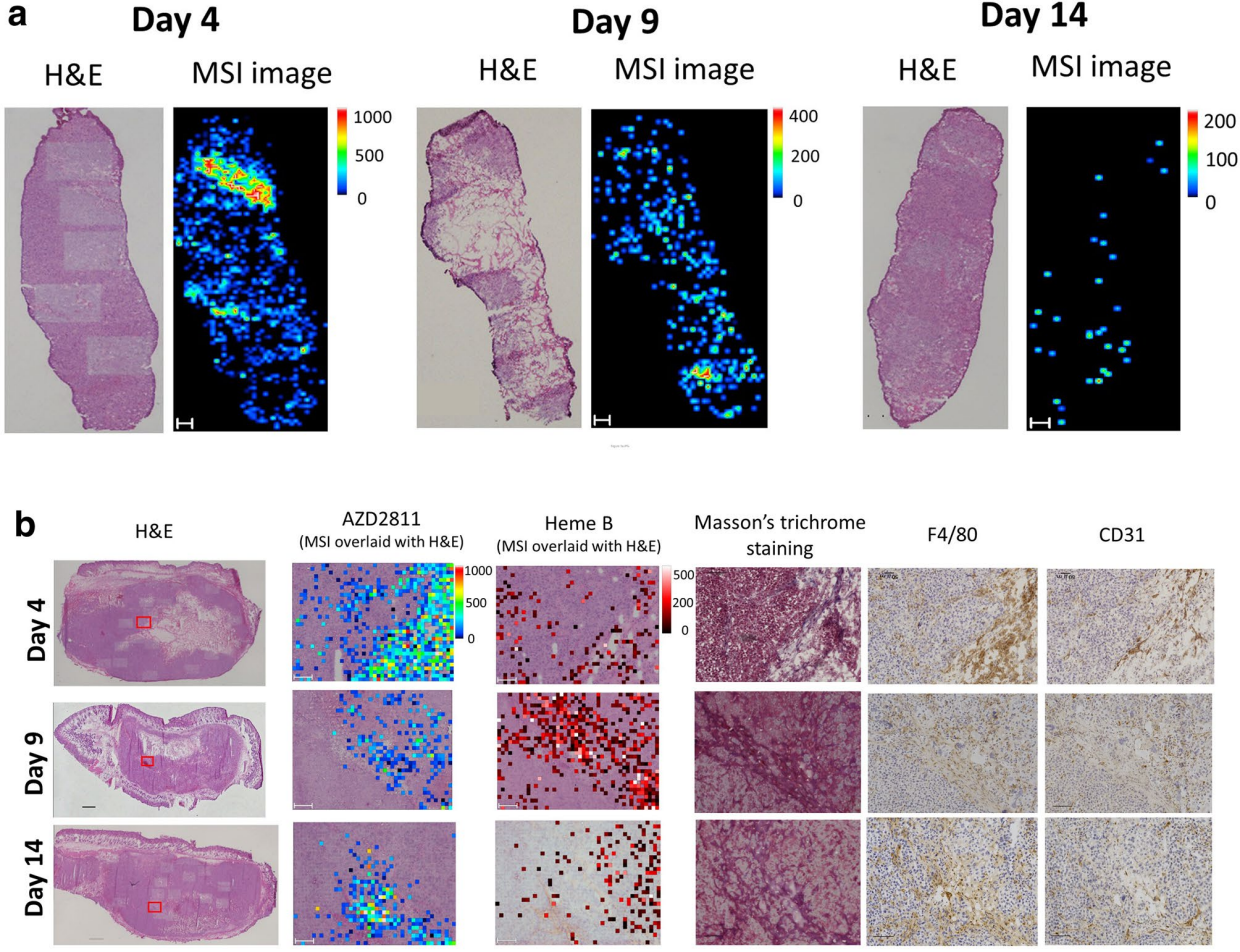
AZD2811 distribution in PC14 biopsy specimens and high-spatial-resolution MSI images of PC14 tumors. (**a**) H&E and AZD2811 MSI images of PC14 tumor biopsy specimens on days 4, 9, and 14. Similar to the whole tumor, AZD2811 (*m/z* 130.12 ± 0.05 Da) was distributed diffusely and heterogeneously in biopsy specimens. Scale bars: 200 μm. (**b**) Representative images of regions of interests (ROIs) for PC14 tumors analyzed at a high spatial resolution (20 μm) under × 20 magnification and the corresponding ROIs for blood distribution depicted by heme B (*m/z* 557.16 ± 0.05 Da) MSI. Collagen (Masson’s trichrome staining), macrophage (F4/80) and vessel (CD31) were stained using consecutive sections**.** Scale bars: 1000 μm for whole tumors and 100 μm for ROIs. Color bar of MSI images: absolute intensity (a.u.: arbitrary unit).

We took three repeat biopsies along the long axis of the PC14 tumor using a 14-gauge core needle. The distribution of AZD2811 in the biopsy specimens was analyzed by MSI. Similar to the results observed with whole tumors, AZD2811 exhibited a heterogeneous distribution, with higher signal intensity on day 4. The AZD2811 signal intensity decreased over time in the biopsy specimens, such that only limited AZD2811 was detected at various hotspots by day 14 (Fig. 3a).

To obtain a more comprehensive visualization of drug distribution, we conducted high spatial resolution MSI analysis of several regions of interests (ROIs) set as 1408-pixel squares analyzed at a spatial resolution of 20 μm. Consecutive sections were stained or examined for histologic factors that could affect the intratumoral distribution of nanomedicines, including vessels, blood distribution, macrophages, and collagen. Heme B, a marker of red blood cells, was analyzed using MSI to assess the blood distribution. The collagen was evaluated by Masson’s trichrome staining. Macrophage and blood vessels were evaluated by immunohistochemical staining with F4/80 and CD31, respectively. By comparing MSI images with the corresponding ROIs in consecutive sections, AZD2811 was noted to distribute in regions rich in collagen where infiltration of macrophages was also observed in PC14 whole-tumor specimens (Fig. 3b).

## Discussion

In this study, we demonstrated the applicability of MSI for evaluating nanoparticle-based drug distribution and PK in tumors. MSI is also applicable for analysis of biopsy samples, indicating that it is also suitable for evaluating intratumoral PK of nanomedicines in clinical practice.

The PK of nanomedicines and conventional small-molecule drugs in tumors differ markedly^26^. Many factors affect the entry, distribution, and clearance of nanoparticles in tumors. Various properties of the nanoparticles themselves (size, charge, surface modifications, controlled release formulations) are critical parameters that have been extensively investigated^27,28^. However, pathophysiologic factors in tumors such as the EPR effect, the stroma, lymphatic system, and mononuclear phagocyte system can also significantly affect the intratumoral behavior of nanoparticles. The complex PK in of nanoparticles in tumors and insufficient PK research data have hindered the translation of nanoparticle-based drug into clinical use^29^.

MADLDI-MSI is a novel, label-free imaging method that has been used to study the PK of small-molecule drugs. In the present study, we applied this novel approach to evaluate the distribution of nanoparticle-formulated AZD2811. AZD2811 nanoparticle is a PLA-PEGylated nanoparticle in a size of about 100 nm, which is below the cutoff size (< 400 nm) of macromolecules that can extravasate into the tumor through passive targeting mechanism via enhanced permeability and retention (EPR) effect^30^. By visualizing AZD2811 in three cell line xenograft tumors (MDA-MB231, HCC1954, and PC14) after AZD2811 nanoparticle administration, MSI revealed a very heterogeneous intratumoral drug distribution and intensity decreased over time. The drug might undergo release from the nanoparticle locally then metabolizing to other products in tumor, or diffusing into the local circulatory system to be removed from the tumor. By comparing MSI with H&E images, we found that AZD2811 was distributed in a more-concentrated manner in some tumor regions and less-concentrated manner in other regions, even in areas indicated by H&E staining to be relatively homogeneous. Many intra-tumoral factors could influence nanoparticle delivery in tumor^27^. Blood flow, vascularization, the permeability of vessels^31–34^, macrophages^35–37^, and organization of collagen^31,38^ have been reported as to be the main affecting factors in nanoparticle intra-tumoral distribution. These heterogeneous organization of histological factors, which differ not only by tumor types but also in the same tumor, might be the reasons for the heterogeneous drug distribution observed in this study.

Considering the limited number of animals used in the present study, we were not able to conduct statistical analyses and only compared images in the corresponding regions. In PC14 whole tumors, AZD2811 exhibited higher signal intensity in locations rich in collagen and macrophage infiltration. The results suggest that the two factors may play important roles in nanoparticle distribution. Previous studies have shown that the collagen network can affect the penetration of nanotherapeutics^38^, and macrophage is assumed as an off-target depletion site for nanoparticles^5,9^. Macrophages have been reported to be related to deposition and breakdown of various types of collagens in tumor microenvironment^39^, indicating a mutual connection for the two factors in nanoparticle distribution.

The AZD2811 visualized by MSI was assumed to include both released and potentially releasable drug in nanoparticles. To validate the MSI method, we plotted the mean signal intensity determined by MSI versus the AZD2811 concentration per mm^2^ as determined by LC–MS/MS. The MSI signal intensity exhibited good linearity with the drug concentration in sections of the same tumor type. The correlation decreased when including all samples of three tumor types. Normalization with an isotopic label compound as an internal standard is usually used. However, the normalization with D5-AZD2811 as IS did not compensate for the disparities between tumor types. From the MSI image of IS, the signal levels of IS were found different among three tumors indicating different ion suppression effects between tumor types. In tissues with higher ion suppression effect, more “dead pixels” (no detection of IS) will occur and increase the bias in our pixel-by-pixel IS normalization. We attempted the tissue extinction coefficient (TEC) by dividing the on-tissue IS mean intensity to the off-tissue mean intensity. AZD2811 mean intensities after normalization with TEC showed an improved correlation to the drug concentration with all samples, indicating TEC is a useful scaling factor to compare different tumor types. Considering the small sample size of this study, further research with a larger sample set is needed.

In clinical research, needle biopsies are often collected; however, they are rarely used for analyzing the PK of drugs in tumors. In the present study, we evaluated the applicability of biopsy specimens for MSI analysis of nanoparticle-formulated drugs. Three repeat biopsies were collected from the same PC14 xenograft tumor. The biopsies revealed a heterogeneous intratumoral distribution of AZD2811 and a time-dependent reduction in intensity that was consistent with that observed in the whole PC14 tumor. Our results thus confirm the clinical applicability of MSI to the analysis of biopsy specimens, and we expect that it will be used in future development of nanoparticle-based drugs.

In terms of limitations of this study, we were not able to visualize nanoparticle adducts, which often have high molecular weights and are difficult to be tracked at our MSI platform. This drawback could be overcome in the future by combining other MSI platforms to trace the nanoparticle constituents (PLA polymer, etc.) that have a high molecular weight. Lack of control group of free AZD2811 is also a limitation of this study that we focused merely on nanoparticle formulated drug intra-tumoral visualization as our primary aim. By referring to the pharmacokinetic profile and MSI of free AZD2811 in a previous study^12^, in which AZD2811 was undetectable at 24 h after dosing in colon adenocarcinoma (SW620) xenograft tumor, we presumed that drug concentration might be low in the tumor at the time points (day 4, day 9 and day14) in this study. Evaluation of the free drug simultaneously is needed when the potential advantages of the nanoparticle are concerned in the future study.

In addition, the visualized AZD2811 distribution did not allow for distinguishing released drug from encapsulated drug. Drug metabolites could be used as a surrogate of released drug from nanoparticle. Several metabolites of AZD2811 in vivo have been reported in a previous study^40^. Monitoring drug metabolites distribution is needed in the future study, which would help determine released drug. In the present study, quantification of the total AZD2811 in plasma and tumor tissues make it possible to link the results of MSI to LC-MSMS. The good correlation with total AZD2811 concentration indicates that MSI can provide information on local potential drug exposure within the same tumor type. Further confirmation will require analysis of a larger set of samples sufficient for statistical analyses and in more clinically relevant animal models, such as patient-derived xenografts.

In conclusion, our study demonstrated an imaging strategy suitable for evaluating the distribution of nanoparticle-based drugs in tumors as well as the evaluation of tumor needle biopsy specimens. This imaging strategy can also be extended to other small-molecule nanoparticle-encapsulated medicines. By combining histologic analysis with MSI, drug distribution can be correlated with histologic factors that could affect the intratumoral distribution of a drug, with the ultimate purpose of selecting patients who might benefit from better delivery and response in clinical nanomedicine.

## Methods

### Materials

AZD2811 nanoparticles were provided by AstraZeneca. AZD2811 (cat no. SML0268) and α-cyano-4-hydroxycinnamic acid (α-CHCA) were purchased from Sigma-Aldrich (St. Louis, MO, USA). 2,5-Dihydroxybenzoic acid (DHB), formic acid (FA), trifluoroacetic acid (TFA), acetonitrile, methanol, 2-propanol, Mayer’s hematoxylin solution, and 1% eosin Y solution for H&E staining were purchased from Wako Pure Chemical Industries Ltd. (Osaka, Japan). Masson’s trichrome staining kits were purchased from Polysciences, Inc. (Warrington, PA, USA). Reagents for immunohistochemistry were purchased from Cell Signaling Technology (Beverly, MA, USA), Abcam (Cambridge, UK), BD Biosciences (Franklin Lakes, NJ, USA), and Dako (Santa Clara, CA, USA).

### Cell lines

MDA-MB231 human triple-negative breast cancer cells (adenocarcinoma) were cultured in DMEM with 10% fetal bovine serum. HCC1954 Her2-positive breast cancer cells (ductal carcinoma) and PC14 lung adenocarcinoma cells were cultured in RPMI medium with 10% fetal bovine serum at 37 °C. MDA-MB231 and HCC1954 cells were purchased from the American Type Culture Collection (Rockville, MD), and PC14 cells were provided by the RIKEN BRC through the National Bio-Resource Project of the MEXT/AMED, Japan.

### Animal experiments

Animal experiments were carried out in full compliance with the Guideline for Animal Experiments (Committee for Animal Experimentation of National Cancer Center, Japan). All experimental animal protocols were approved by the Institutional Animal Ethics Committee of the National Cancer Center (permission nos.: T14-024, T15-019).

Male BALB/c Slc-nu/nu mice (5–6 weeks old; Japan SLC, Inc., Shizuoka, Japan) were used in the experiments. For the drug distribution study, 1 × 10^7^ MDA-MB231HCC1954 cells or 5 × 10^6^ PC14 cells were inoculated into the dorsal subcutaneous tissue (n = 1 at each time point). Tumor development was assessed twice each week using calipers, and mice were randomized (day 0) when the tumor volume reached approximately 200 mm^3^. AZD2811 nanoparticles (25 mg/kg) were intravenously administered on days 1 and 3. Mice were anesthetized with pentobarbital (Kyoritsu Seiyaku Corp., Tokyo, Japan), and tumor and biopsy samples were collected using fine-core disposable, semiauto biopsy needles, 14-gauge × 200 mm (cat no. 010214200; Toray, Tokyo, Japan) on days 4, 9, and 14. All mice were sacrificed after study completion, and blood was collected from the heart. Tumor tissue and biopsy specimens were snap frozen in liquid nitrogen and stored at − 80 °C until analyses.

### MSI

For selecting the optimal matrix, the standard solution of free AZD2811 was prepared at 1000 pg/μL in 50% methanol. Then, the standard solution were mixed (1:1) with 10 mg/mL α-CHCA or DHB in 30% acetonitrile/10% 2-propanol/0.1% TFA (or 0.1%FA or without adding acid). For on-glass and on-tissue spotting, a standard solution of free AZD2811 was prepared in 50% methanol, and AZD2811 nanoparticle standard solution was prepared in ddH_2_O at known concentration, then mixed (1:1) with 10 mg/mL α-CHCA in 30% acetonitrile/10% 2-propanol/0.1% TFA. The spotting volume was 0.1μL per spot.

Snap-frozen tumor tissues were sectioned into slices at 8-µm thickness at − 20 °C in cryostat (Leica CM1950,Tokyo, Japan )and mounted onto indium titanium oxide–coated slide glasses (SI0100N, Mastunami Glass Inc., Ltd., Tokyo, Japan) for MALDI-MSI analysis. α-CHCA (Sigma-Aldrich) was applied to the tissue surface at 250 °C for 8 min using a sublimation apparatus (SVC-700TMSG/7PS80, Sanyu Electron, Tokyo, Japan). Next, 10 mg/mL α-CHCA in 30% acetonitrile/10% 2-propanol/0.1% TFA was sprayed stepwise onto the sections using a sprayer (PS270, GSI Creos Corp., Tokyo, Japan). D5-AZD2811 was added to the matrix solution at a final concentration of 2 μg/mL.

MALDI-MSI analysis was performed in positive mode using iMScope (Shimadzu, Kyoto, Japan), with a resolution of 10,000 at *m/z* 1,000. Mass spectra were obtained over the mass ranges of 50–520 Da and 50–525 Da for AZD2811 and D5-AZD2811, respectively. The raster size was set at 50 μm, with 3 × 50 laser shots for each spot. Laser power (Nd:YAG, WL: 355 nm) was optimized at 49 for AZD2811 and 51 for D5-AZD2811. The MS/MS transitions for AZD2811 and D5-AZD2811 were *m/z* 508.24 → 130.12 with collision energy of 66 V and *m/z* 513.27 → 135.15 with collision energy of 62 V, respectively. The MS/MS transition for heme B was *m/z* 616.18 → 557.16. For high spatial resolution analyses, the raster size was set at 20 μm. Imaging data were analyzed using Imaging MS Solution ver. 1.20 (Shimadzu, Japan) and Biomap software (Novartis, Basel, Switzerland).

### LC–MS/MS analysis

To measure the plasma concentration of total AZD2811, 20 µL of plasma was mixed with 10 µL of D5-AZD2811 solution (1 µg/mL), after which 100 µL of methanol was added and vortexed for protein precipitation. D5-AZD2811 was used as an internal standard. The sample was then centrifuged at 12,000×*g* for 10 min. The supernatant was diluted in 0.1% FA/10 mM ammonium formate/90% acetonitrile and centrifuged at 12,000×*g* for 10 min, filtered (0.45 µm, MSRLN0410, Merck Millipore Ltd.) at 500×*g* for 5 min, and then used for analysis. For tumor tissues, two consecutive tumor sections (before and after the section for MSI) were homogenized in 100 μL of methanol to precipitate protein, followed by the addition of 100 μL of H_2_O. The tissue extract was diluted in 0.1% formic acid/10 mM ammonium formate/90% acetonitrile and used for analysis after filtering (0.45 µm, MSRLN0410, Merck Millipore Ltd.) at 500×*g* for 5 min.

A Discovery HS F5 HPLC column (5 μm particle size, 15 cm × 4.6 mm, Sigma-Aldrich) was used for the separation of AZD2811. The column temperature was set at 40 °C. Mobile phase A consisted of 0.1% FA/10 mM ammonium formate, and mobile phase B consisted of 0.1% FA/10 mM ammonium formate/90% acetonitrile. The rinse solution was 0.1% FA/10 mM ammonium formate/80% acetonitrile. The flow rate was 1 mL/min, with an injection volume of 10 µL. The retention time for both AZD2811 and D5-AZD2811 was 3.3 min. Samples were analyzed on a QTRAP4500 mass spectrometer (AB SCIEX, Framingham, MA, USA) with electrospray ionization in the positive mode. The selected reaction monitoring transition was *m/z* 508.07 → 130.10 for AZD2811 and *m/z* 513.27 → 135.15 for D5-AZD2811. The optimized parameters were as follows: ion source temperature, 650 °C; curtain gas, 30; nebulizing gas (GS1), 40; turbo-ion spray gas (GS2), 70; ion spray voltage, 5500 V; collision energy, 31 V. Data were analyzed using Analyst version 1.6.1 software (AB SCIEX).

### Histologic analysis

Frozen tumor sections were stained with hematoxylin and eosin (Wako Pure Chemical Industries Ltd.). Collagen was stained using a Masson’s trichrome staining kit (25088-100, Polysciences, Inc.). For immunochemical staining, frozen tumor sections were fixed in 10% formalin for 10 min at room temperature. After washing with 1 × TBS, sections were incubated with 0.3% H_2_O_2_ in methanol at room temperature, followed by washing in 1 × TBS. To inhibit non-specific staining, sections were incubated in serum-free protein block solution (X0909, Dako) for 15 min at room temperature. The sections were then incubated with primary antibodies: rat anti-mouse CD31 monoclonal antibody (BD557355, BD Biosciences, 1:100) or rat anti-mouse F4/80 monoclonal antibody (MCA497, Bio-Rad, 1:100). After washing with 1 × TBS, signal stain boost IHC detection reagent (anti-rabbit, Cell Signaling Technology) or rabbit anti-rat IgG H&L (horseradish peroxidase conjugated) (ab6734, Abcam, 1:500) was added, and sections were incubated at room temperature for 30 min. After reaction with diaminobenzidine (8059S, Cell Signaling Technology), sections were counterstained with hematoxylin. A BZ-X710 microscope (Keyence, Osaka, Japan) was used for histologic observation and evaluation.

## Supplementary information

Supplementary file1
