# Supplementary material for: Visualization of the distribution of nanoparticle-formulated AZD2811 in mouse tumor model using matrix-assisted laser desorption ionization mass spectrometry imaging
Source: Sci Rep. 2020 Sep 23;10:15535. doi: 10.1038/s41598-020-72665-5 (PMC7511311; doi:10.1038/s41598-020-72665-5)
Supplement: Supplementary file 1 — Supplementary file1 [file 41598_2020_72665_MOESM1_ESM.docx]

**Visualization of the distribution of nanoparticle-formulated AZD2811 in mouse tumor model using matrix-assisted laser desorption ionization mass spectrometry imaging**

Shoraku Ryu^1^, Mayu Ohuchi^1,2^, Shigehiro Yagishita^1^, Tatsunori Shimoi^3^, Kan Yonemori^3^ Kenji Tamura^3^, Yasuhiro Fujiwara^3^, Akinobu Hamada*^1, 2^.

^1^ Division of Molecular Pharmacology, National Cancer Center Research Institute, Tokyo, Japan.

^2^ Department of Medical Oncology and Translational Research, Graduate School of Medical and Pharmaceutical Sciences, Kumamoto University, Kumamoto, Japan.

^3^ Department of Breast and Medical Oncology, National Cancer Center Hospital, Tokyo, Japan

*Corresponding Author: Akinobu Hamada, PhD

E-mail: akhamad@ncc.go.jp

Division of Molecular Pharmacology, National Cancer Center Research Institute, Tsukiji 5-1-1, Chuo-ku, Tokyo 104-0045, Japan

Phone: +81-3-3542-2511; Fax: +81-3-3542-3815

**Supplementary data**

**Figure S1**

**(a)** Select the matrix and acid. Standard solution of free AZD2811 at concentration of 500 pg/µL (0.1µL per spot) was spotted on slide glass (n=3). Detection AZD2811 sensitivity (m/z 508.24±0.05Da) of two types of matrix (α-CHCA and DHB) and acid (FA and TFA) were compared. Error bar: standard deviation (SD) of three spots.

**(b)** Confirm the specificity of precursor ion (m/z 508.24±0.05Da) and product ion (m/z 130.12±0.05Da) of AZD2811 by spotting free AZD2811 standard solution (100pg/spot) on blank tumor tissue (untreated MDA-MB231 tumor). An endogenously derived peak (m/z 508.30) was near the target peak of AZD2811 (m/z 508.24) at MS measurement. No peak at m/z 130.12 is detected in a blank spot on blank tumor, indicating a good specificity at MS/MS measurement. Scale bar: 200 μm.

**(c)** Confirm MSI analysis of nanoparticle formulated AZD2811 by spotting standard solution on slide glass. The standard solution spots were prepared at concentrations of 10 pg/spot, 20 pg/spot, and 50 pg/spot (n=3). The total intensity per spot in MSI analysis (*y*-axis) were compared to the drug concentration (*x-* axis). Error bar: standard deviation (SD) of three spots.

**Figure S2**

**(a)** The correlation between the signal responses in MSI analysis and drug concentration analyzed by LC-MS/MS in MDA-MB231, HCC1954, and PC14 whole tumor slices on day 4, day 9, and day14. The AZD2811 total concentration per tissue area (pg/mm^2^) was quantified by LC-MS/MS from 2 consecutive tumor slices adjacent to the section for MSI and plotted on the *x*-axis. AZD2811 mean signal intensity per tissue area in MSI images was correlated to LC-MS/MS according to each tumor type, or all samples (the upper panel). Normalization of AZD2811 mean intensity with IS or TEC (the lower panel) were also compared (the lower panel).

**(b)** MSI images of D5-AZD2811(m/z 135.15±0.05Da) in tumor sections.

**Figure S1**


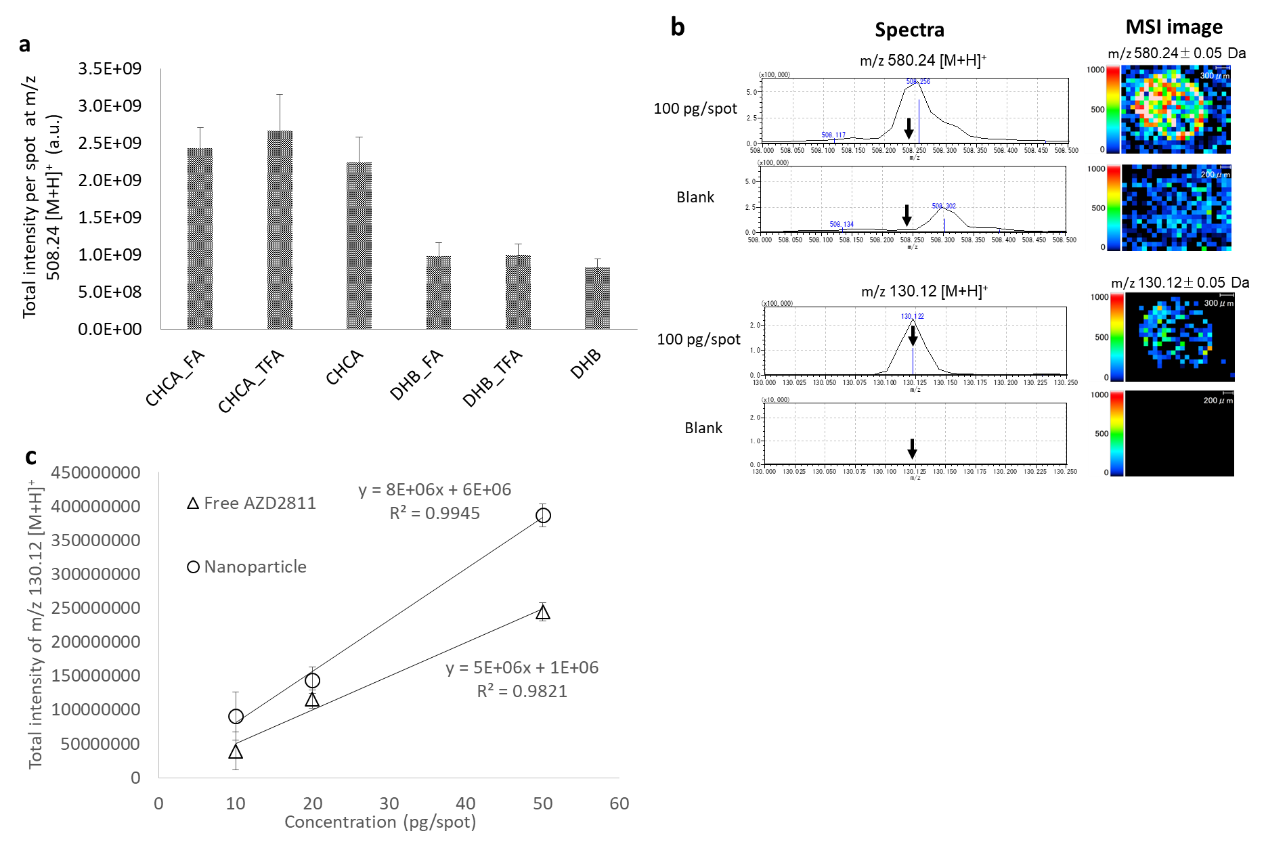


**Figure S2**

**
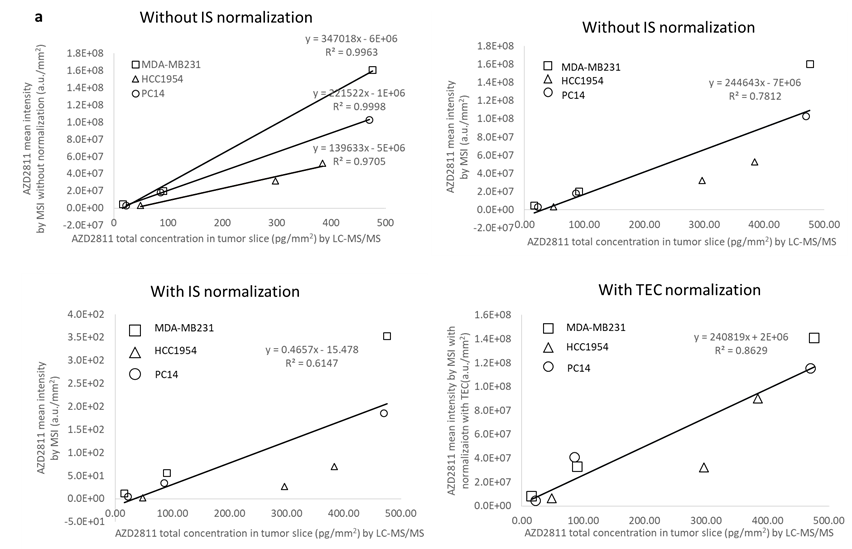
**

**
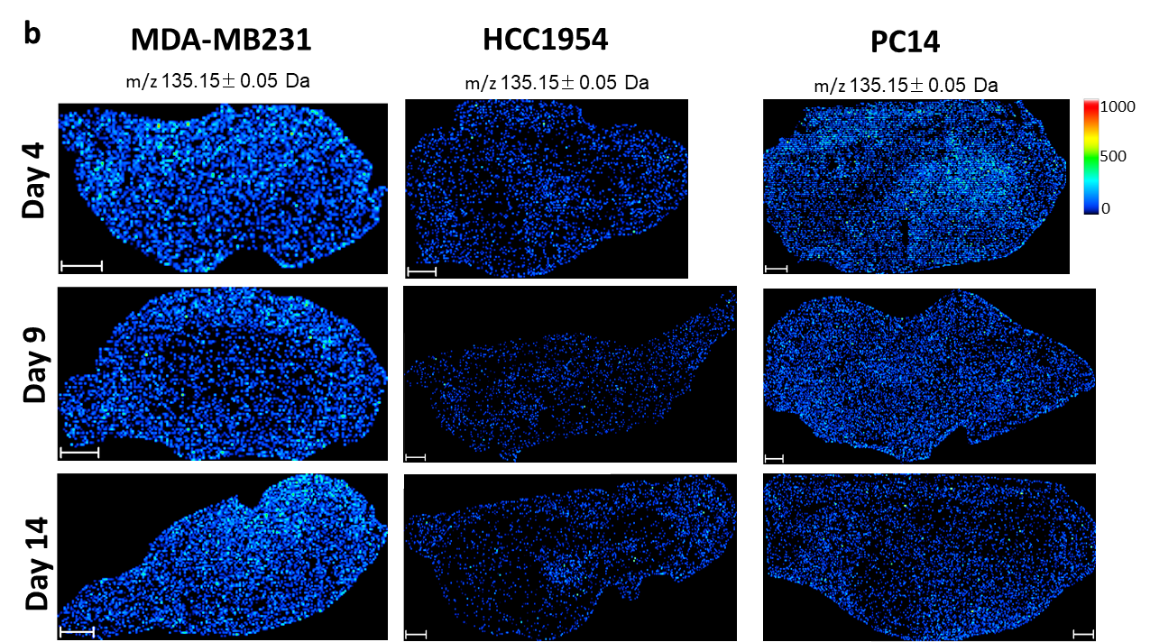
**

**Table S1**

The mean intensity of AZD2811 for nanoparticle standard spots on 2 consecutive untreated MDA-MB231 tumor section.


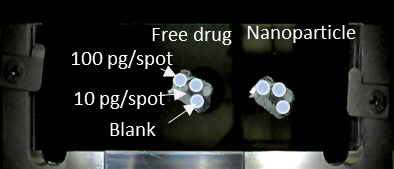


| Total drug concentration (pg/ spot) | Total intensity(a.u.) | |
| --- | --- | --- |
|  | Free AZD2811 spot on tumor tissue (n=1) | AZD2811 nanoparticle spot on tumor tissue (n=1) |
| 100 | 1.3E+08 | 3.7E+07 |
| 10 | 8.2E+06 | 4.1E+06 |
| Blank | N.D. | N.D. |

**Table S2**

Optimized conditions for mass spectrometry imaging at iMScope system.

| Instrument | iMScope（SHIMADZU） |
| --- | --- |
| Polarity | positive |
| Laser | N2（WL：337 nm） |
| Matrix | 10mg/ml α-CHCA（α-cyano-4-hydroxycinnamic acid） |
| Matrix solution | H_2_O/Acetonitrile/2－Propanol/TFA（60:30:10:0.1） |
| Matrix treatment | Two steps method(sublimation+ manual spraying) |
| Scan range | m/z 50-520 (AZD2811)  m/z 50-525 (D5-AZD2811) |
| Selected Reaction Monitor | m/z 508.2 → m/z 130.1 (AZD2811)  m/z 513.28→m/z 135.15 (D5-AZD2811) |
| Mass Width (Da) | 1.5 |
| CE (a.u.) | 66 (AZD2811) 62 (D5-AZD2811) |
| LP (%) | 49 (AZD2811) 51 (D5-AZD2811) |

**Table S3**

Optimized parameters for LC-MS/MS analysis

HPLC

| Column | Discovery HS F5, 5 μm particle size, 15 cm × 4.6 mm | |
| --- | --- | --- |
| Solution A | 10mM Ammonium Formate/ FA (100:0.1 (v/v)) | |
| Solution B | 100mM Ammonium Formate/ACN/FA (10:90:0.1 (v/v/v)) | |
| Gradient | Time (min) | B. Conc. (%) |
|  | 4.00 | 90 |
|  | 4.10 | 100 |
|  | 7.00 | 100 |
|  | 7.10 | 90 |
|  | 10 | 90 |
| Flow rate | 1 mL/min | |
| Injection volume | 10 µL | |
| Retention Time (min) | 3.32 | |

MRM

| Compound | Ionization mode | Polarity | Precursor ion  (m/z) | Product ion  (m/z) | Curtain Gas (CUR) | Collision Gas (CAD) | IonSpray Voltage (IS) | Temperature (TEM) | Ion Source Gas 1 (GS1) | Ion Source Gas 2 (GS2) |
| --- | --- | --- | --- | --- | --- | --- | --- | --- | --- | --- |
| AZD2811 | ESI | Positive | 508.070 | 130.100 | 30 | 8 | 5500 | 650 | 40 | 70 |
| D5-AZD2811  (IS) | ESI | Positive | 513.278 | 135.153 |  |  |  |  |  |  |

Table S4

The calculated tissue extinction coefficient (TEC) in each tumor section by dividing the IS mean intensity of tumor tissue to off-tissue region.

|  | IS mean intensity_tumor tissue | IS mean intensity_off-tissue region | TEC |
| --- | --- | --- | --- |
| PC14_Day 4 | 115564 | 129683 | 0.89 |
| PC14_Day 9 | 68998 | 153479 | 0.45 |
| PC14_Day 14 | 49245 | 72898 | 0.68 |
| MB231_Day 4 | 174146 | 152749 | 1.14 |
| MB231_Day 9 | 103888 | 170064 | 0.61 |
| MB231_Day 14 | 132160 | 229718 | 0.58 |
| HCC1954_Day 4 | 40960 | 70501 | 0.58 |
| HCC1954_Day 9 | 22110 | 22237 | 0.99 |
| HCC1954_Day 14 | 31868 | 62205 | 0.51 |
